# Diagnostic performance of ultrasound-based artificial intelligence for predicting key molecular markers in breast cancer: A systematic review and meta-analysis

**DOI:** 10.1371/journal.pone.0303669

**Published:** 2024-05-31

**Authors:** Yuxia Fu, Jialin Zhou, Junfeng Li

**Affiliations:** 1 Department of Ultrasound, Dianjiang People’s Hospital of Chongqing, Chongqing, China; 2 Department of Oncology, Dianjiang People’s Hospital of Chongqing, Chongqing, China; University of Pisa, ITALY

## Abstract

**Background:**

Breast cancer (BC) diagnosis and treatment rely heavily on molecular markers such as HER2, Ki67, PR, and ER. Currently, these markers are identified by invasive methods.

**Objective:**

This meta-analysis investigates the diagnostic accuracy of ultrasound-based radiomics as a novel approach to predicting these markers.

**Methods:**

A comprehensive search of PubMed, EMBASE, and Web of Science databases was conducted to identify studies evaluating ultrasound-based radiomics in BC. Inclusion criteria encompassed research on HER2, Ki67, PR, and ER as key molecular markers. Quality assessment using Quality Assessment of Diagnostic Accuracy Studies (QUADAS-2) and Radiomics Quality Score (RQS) was performed. The data extraction step was performed systematically.

**Results:**

Our meta-analysis quantifies the diagnostic accuracy of ultrasound-based radiomics with a sensitivity and specificity of 0.76 and 0.78 for predicting HER2, 0.80, and 0.76 for Ki67 biomarkers. Studies did not provide sufficient data for quantitative PR and ER prediction analysis. The overall quality of studies based on the RQS tool was moderate. The QUADAS-2 evaluation showed that the studies had an unclear risk of bias regarding the flow and timing domain.

**Conclusion:**

Our analysis indicated that AI models have a promising accuracy for predicting key molecular biomarkers’ status in BC patients. We performed the quantitative analysis for HER2 and Ki67 biomarkers which yielded a moderate to high accuracy. However, studies did not provide adequate data for meta-analysis of ER and PR prediction accuracy of developed models. The overall quality of the studies was acceptable. In future research, studies need to report the results thoroughly. Also, we suggest more prospective studies from different centers.

## 1. Introduction

Breast cancer (BC) is the most prevalent malignancy worldwide (11.7% of all cancer cases in 2020), and it is acknowledged as one of the leading causes of cancer-related mortality (6.9% of all cancer-related deaths in 2020), according to GLOBOCAN reports [1]. Traditional predictors such as tumoral and pathological properties are well-established in BC but could not classify the patients comprehensively regarding intra- and inter-patient heterogeneity. Therefore, developing more reliable and precise predictors could facilitate categorizing patients into subtypes with distinct characterizations [2, 3]. Molecular biomarker profiling of BC, determined by gene expression experiments, could classify patients into different subtypes based on different expression levels of these biomarkers in each subtype [4]. This classification revealed that BC subtypes have distinct diagnostic, therapeutic, and prognostic features that can significantly aid escalation/de-escalation options. Estrogen receptor (ER), progesterone receptor (PR), human epidermal growth factor receptor 2 (HER2), and Ki67 are the main molecular biomarkers in patients of BC [5]. Evidence has shown that hormone receptors such as ER and PR are associated with a good prognosis, and PR/ER-negative patients have a poorer prognosis [6]. Patients with positive HER2, a transmembrane tyrosine kinase receptor, have been shown to have a poorer prognosis and more aggressive features of the tumor [7]. Also, Ki67, as an expressed antigen in cell cycle phases, has a positive correlation with tumor aggressiveness and proliferative activity, hence poorer prognosis and recurrence in patients of BC [8]. ER, PR, HER2, and Ki67 are evaluated mainly by immunohistochemical (IHC) analysis after biopsy or surgically excised samples. However, this method is not only an invasive and time-consuming procedure, but a single local tumor specimen only sometimes captures the status of these biomarkers in the whole tumor. Therefore, it is urgently needed to establish a non-invasive, easy-to-do, and more representative method [9]. Current limitations of molecular paradigms to classify BC patients could be overcome by the new era of artificial intelligence (AI) algorithms. There are different AI methods, of which AI-powered radiomics and deep learning are the two main approaches. The AI-powered Radiomics method is an umbrella term for the extraction of quantitative and reproducible information from medical images such as ultrasonography (US), computed tomography (CT) scan, and magnetic resonance imaging (MRI), to develop decision support models recognizing complex patterns of images that are not visible by human naked eyes [10]. On the other hand, deep learning (DL), as a convolutional neural network-based algorithm, can also assist clinical decision-making by self-education of complex patterns of diagnostic images, bypassing the manual extraction of imaging features [11].

Therefore, developing a non-invasive AI-based decision-making system using routine imaging modalities (US, CT, or MRI) before any invasive procedure is necessary to predict the essential molecular biomarkers in BC tissue. Herein, we searched databases and designed this systematic review and meta-analysis of current data on the accuracy of AI-based methods for predicting key molecular markers in BC and evaluating the quality of studies.

## 2. Methods

This systematic review and meta-analysis is conducted in line with the Preferred Reporting Items for Systematic Review and Meta-Analyses of Diagnostic Test Accuracy Studies (PRISMA-DTA) guidelines [12].

### 2.1. Search strategy

Two independent observers (YF, and JZ) searched databases (PubMed, EMBASE, and Web of Science) from inception to October 30, 2023. After duplicate removal, YF and JZ independently screened the title and abstract of studies to evaluate the eligibility. Then, the full text of the remaining studies was screened according to the study selection criteria. The reference list of included studies was searched to identify possible eligible studies. Any disagreement during the database search was discussed and resolved by consultation.

### 2.2. Inclusion criteria

We selected the studies for review if they met the following inclusion criteria based on PICO (Population, Index test, Comparator, and Outcome) elements:

1. Population: patients diagnosed with BC.
2. Index test: using AI-based methods to predict the status of the key molecular biomarkers (HER2, Ki67, PR, and ER) in patients.
3. Comparator: using biopsies or surgically excised samples to determine the status of the molecular biomarkers.
4. Outcome: Studies with enough data to predict the status of key molecular biomarkers in BC patients based on US images before surgical procedures (biopsy, excisional biopsy, surgery).

### 2.3. Exclusion criteria

We excluded the records that met the following exclusion criteria:

1. Studies incompatible with the PICO criteria mentioned above.
2. Reviews (narrative, systematic, and meta-analysis), case reports, conference abstracts, comments, and letters to editors.
3. Articles in a non-English language.
4. Studies not using AI-based methods.

### 2.4. Data extraction

We used standardized forms to review the included studies. Two independent reviewers (YF, and JZ) extracted the following data upon availability: The first author’s name, year of publication, type of study, country, name, and number of patient selection centers, aim of study, patients’ information (patient number, gender distribution, mean age), AI method, type of US, whether contrast agent is used or not. For radiomics studies, we extracted the following data specifically: segmentation method (automatic manual or semi-automatic), segmentation dimension and software, features number, type of extracted features, feature extraction software, feature selection methods, AI modeling algorithm, and whether the radiomics features are combined with other clinical data. Possible disagreements were discussed and resolved through a consensus.

### 2.5. Quality assessment

We used the “Quality Assessment of Diagnostic Accuracy Studies (QUADAS-2)” tool to evaluate the presence of risk of bias and application concerns in different domains (“patient selection,” “index test,” “reference standard,” and “flow and timing”). Each domain assigned yes, no, or unclear for the assessment of risk of bias and low risk, high risk, or unclear for assessing applicability concerns. Also, we can tailor the QUADAS-2 signaling questions to our research questions to assess the risk of bias [13]. Moreover, we used the “Radiomics Quality Score (RQS)” system to measure the methodological rigor of the studies using the radiomics method. RQS has 16 items with corresponding scores under the following domains: data selection, medical diagnostic images, feature extraction, exploratory analysis, and model construction [14]. Two independent reviewers (JZ, and ZL) assessed the methodological quality and risk of bias using the QUADAS-2 and RQS tools, and disagreements were resolved by discussion and consultation.

### 2.6. Statistical analysis

We used STATA (version 15; MIDAS module) for the meta-analysis. Sensitivity and specificity with 95% confidence interval (CIs) were calculated for pooling the effect sizes. We plotted the summary receiver operating characteristic curve (SROC) to calculate the area under the curve (AUC). We indicated the pooled effect sizes using the forest plot. A random-effect model was utilized to combine the effect sizes and address the potential heterogeneity of studies. We designed a deeks funnel plot for evaluation of the publication bias. Some studies did not have enough data for meta-analysis. We manually calculated the needed parameters using sensitivity, specificity, and the number of positive and negative classes of each molecular biomarker. The Fagan plot was created to evaluate the clinical utility of the included studies with post-test probability when the pretest probabilities were available.

## 3. Results

### 3.1. Literature search

**Fig 1:**
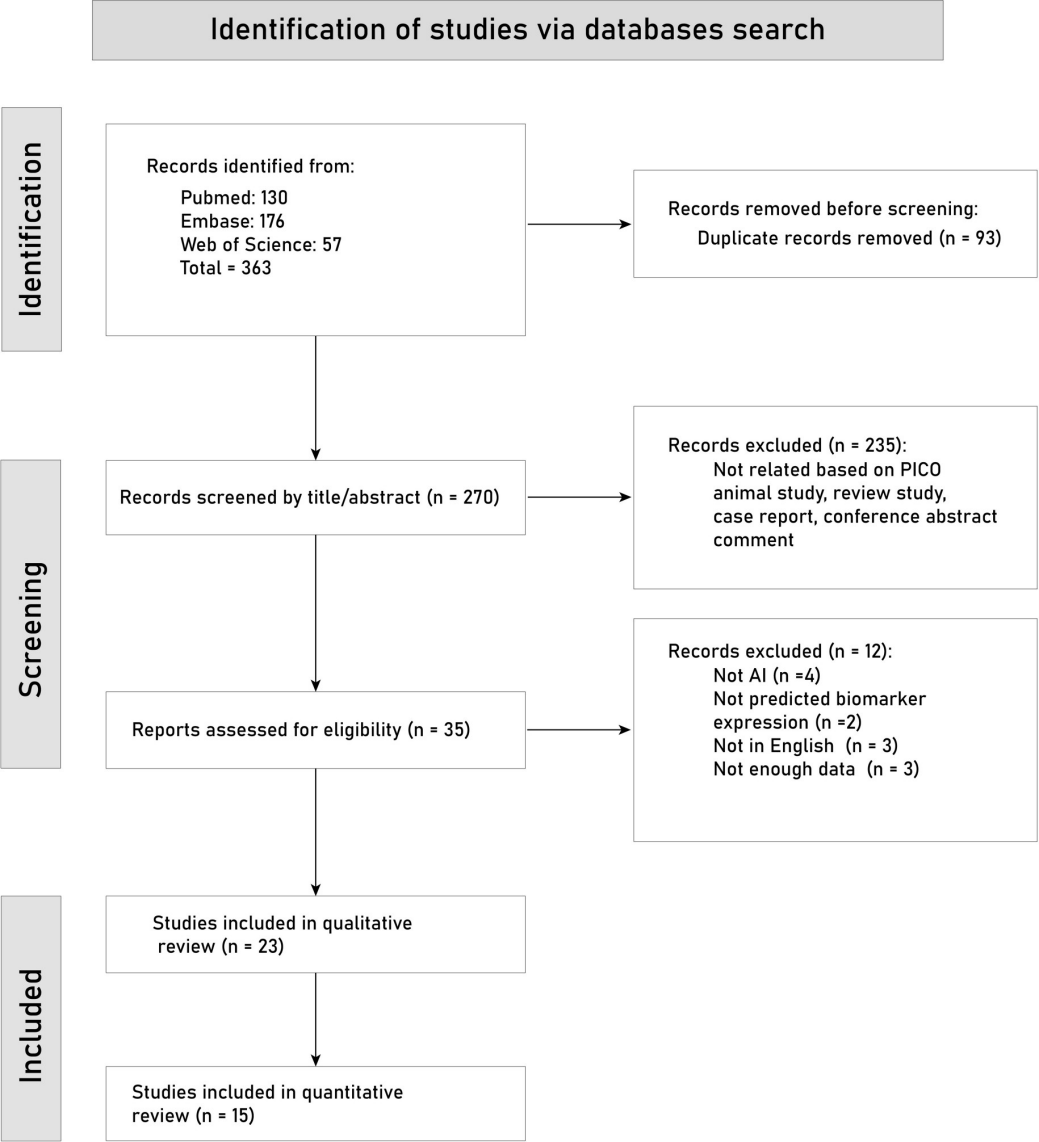
PRISMA flowchart for the included studies.

We outline the study selection process details using the PRISMA flowchart in **Fig 1**. Our initial search identified 363 articles, of which 93 records were duplicates. The titles and abstracts of the 270 records were screened, and 235 articles were removed. After reading through the full text of the remaining 35 articles, twelve more records were removed due to the following reasons: 1) four studies were not about AI methods; 2) two studies did not predict the key molecular biomarkers in BC patients; 3) three studies were not in English; 4) three studies did not provide sufficient data for the review process. Then, we included 23 studies with a total number of 11005 patients for the qualitative review process [15–37].

Of these 23 studies, 15 studies were available for quantitative analysis, and eight studies were not included in the meta-analysis due to inadequate data.

### 3.2. Study characteristics

We showed the general characteristics of 23 included studies in **Table 1**, of which 14 were radiomics. The characteristics of the radiomics workflow are summarized in **Table 2**. Overall, twenty-one (21/23) studies were from China, and the other two were from Romania [33] and Canada [21]. Three studies [15, 25, 33] were prospective (3/23), while the remaining twenty were designed retrospectively (20/23). Regarding AI methods, fourteen studies used radiomics (14/23), six studies used deep learning (6/23), and three studies used machine learning methods (3/23). Three studies [15, 25, 33] retrieved patients from multiple centers (3/23), and the remaining twenty studies were from only one center (20/23). The overall number of patients was 11.005, with a mean age of 52.7 years. However, two studies [20, 23] did not provide the mean age of subjects. One study [36] included men BC; the remaining studies were from the female population. Regarding radiomics studies (14/23), we investigated different steps of radiomics workflow, including segmentation, feature processing, and modeling. Three studies [19, 32, 33] used the automatic/semiautomatic method to delineate the region of interest (ROI). The remaining 19 studies were segmented manually. Only one study [31] did not specify the segmentation method. The open-source ITK-SNAP software was the most commonly used tool for the segmentation process (5/14), and it was utilized in five studies [22, 28, 34, 36, 37]. Two studies used deep learning (2/14) for the segmentation process. Time-intensity curve software, GNU image manipulation program (GIMP), 3D-Slicer, and Darwin Research Platform were used once in the studies. Three studies [25, 31, 35] did not mention the segmentation software. Most studies extracted the shape, first-order, second-order, and wavelet radiomics features regarding the feature processing step. The PyRadiomics package was the most utilized tool to extract the features from ROIs. The extracted features in studies ranged from 86 to 5234. Among these features, the most robust ones should be selected, and different methods are utilized in studies to select them. The Least Absolute Shrinkage and Selection Operator (LASSO) regression was the most commonly used method among studies. Then, the selected features are fitted into a machine learning algorithm to train the data. The logistic regression (LR) algorithm was used in nine studies (9/14) for the model construction.

**Table 1:** General information of the studies.

Author	Study type	Centers	Country	Patients					AI Method	US type	RQS score
				Total	Patients number	Male	Female	Mean age			
Bo-Yang Zhou 2021	Pro	3	China	818	818 BC in 807 P Training:545 in 534 Int validation:85 Test A:93 Test B:95	0	818	57.75	ML	CUS CDUS USE	-
Fang Chen 2020	Ret	1	China	119	119 P Training:80 Test:29	0	119	57	ML	CEUS	-
Fang Chen 2023	Ret	1	China	335	335 P ratio 3:1	0	335	56	DL	CUS CEUS	-
Hao Cui 2020	Ret	1	China	263	263	0	263	49.63	Radiomics	B-mode CDUS USE	6
Hao Cui 2023	Ret	1	China	415	415 P Train: 224 Test:96 Independent valid:95	0	415 P	52.1	Radiomics + DL	CUS	9
Ioana Bene 2022	Pro	1	Romania	72	72	0	72	56.5	Radiomics	CEUS	18
Jiangfeng Wu 2022	Ret	1	China	284	284 P Train:198 Test:86	0	284	53.6	Radiomics	-	14
Jia-wei Li 2022	Ret	1	China	252	252 P	0	252	50.9	Radiomics	-	12
Jinjin Liu 2022	Ret	1	China	324	328L in 324P Train:230 Test:98	1	232	53.2	Radiomics	USE CUS	11
Linyong Wu 2021	Ret	1	China	116	116 P ratio 7:3	0	116	48.8	Radiomics	-	13
Meng Jiang 2020	Ret	3	China	2120	2120P Train:1275 Test1: 405 Test2: 440	0	2120	47.6	DL	-	-
Mengwei Ma 2022	Ret	1	China	600	600P Train:450 Test:150	0	600	47.6	ML	-	-
Meng-Yao Quan 2023	Ret	1	China	445	445P Train:357 Test:88	0	445	50	Radiomics + DL	-	11
Mengyun Qiao 2022	Ret	1	China	502	502P Train:400 Test:102	0	502	-	DL	-	-
Romuald Ferre 2023	Ret	1	Canada	88	88 P	0	88	56	Radiomics	CUS	11
Rong Xu 2023	Ret	1	China	342	359L in 342 P Train:251 Test:108	0	342	54.5	Radiomics	CUS	9
Xianyu Zhang 2021	Ret	2	China	2523	2523 P Train:2804L in 1820P Test: 707L in 620P Ext test: 220L 83P	0	2523	-	DL	-	-
Xiaoying Zhuo 2023	Ret	1	China	103	103 P	0	103	52	DL	CUS USE	-
Xuantong Gong 2023	Pro	1	China	119	119 P	0	119 P 120 L	47.8	Radiomics	CUS CEUS	19
Yimin Wu 2023	Ret	1	China	197	200 L Train: 134 Valid: 66	0	197 P 200 L	55.4	Radiomics	CUS	14
Yinghong Guo 2022	Ret	1	China	309	309 Train: 216 Valid: 93	0	309 P	52.8	Radiomics	CUS	15
Yunpei Zhu 2022	Ret	1	China	515	515 Train: 360 Test: 155	0	515 P	55.3	Radiomics	CUS	15
Zilong Xu 2022	Ret	1	China	144	144 P Train: 108 Test: 36	0	144 P	53.5	DL	CUS	-

Pro: Prospective, BC: Breast Cancer, Ret: Retrospective, ML: Machine Learning, CUS: Conventional Ultrasound, CDUS: Collor Doppler Ultrasound, USE: Ultrasound Elastography, P: Patient

**Table 2:** information of radiomics studies.

Author	Segmentation			Features				Modeling		
	Method	Dim	Software	Types	Number	Software	Selection method	AI method	Algorithm	combined
Hao Cui 2020	-	-	-	-	-	-	-	ML	LR	No
Hao Cui 2023	Automatic	2D	python (DL)	First Order second order	86	Pyradiomics	Pearson correlation analysis	ML	LR	Yes
Ioana Bene 2022	Semi-automatic	2D	Time-intensity curve	texture	-	Mazda	Fisher coefficients Mann–Whitney U	ML	LR	No
Jiangfeng Wu 2022	Manual	2D	ITK-SNAP	first order shape second order Wavelet	15 / 788	Pyradiomics	ICC of > 0.70 Kolmogorov-Smirnov test LASSO	ML	LR	Yes
Jia-wei Li 2022	Manual	2D	-	first order shape second order	25 / 1688	-	PCA LASSO	ML	SVM	No
Jinjin Liu 2022	Manual	2D	ITK-SNAP	first order shape second order	8 / 1218	Pyradiomics	ICCs > 0.75 z-score LASSO	ML	LR	Yes
Linyong Wu 2021	Manual	2D	ITK-SNAP	first order shape Wavelet second order	- / 5234	Pyradiomics	Spearman correlation coefcient LASSO	ML	DT	No
Meng-Yao Quan 2023	Automatic	2D	python (DL) YOLO	-	91 + 14	-	-	ML	XGBoost	Yes
Romuald Ferre 2023	Manual	2D	GIMP	first order shape second order	8 / 249	Pyradiomics	t-test	ML	LR	No
Rong Xu 2023	Manual	2D	ITK-SNAP	first order shape second order	16 / 1314	Pyradiomics	ICC > 0.75 Pearson’s coefficients LASSO	ML	SVM	No
Xuantong Gong 2023	Manual	2D	-	-	-	Pyradiomics	mRMR	ML	LR	No
Yimin Wu 2023	Manual	2D	3D-Slicer	shape first order second order wavelet	15 / 1702	Pyradiomics	LASSO	ML	LASSO	Yes
Yinghong Guo 2022	Manual	2D	ITK-SNAP	shape statistics texture wavelet	- / 788	Pyradiomics	ICC > 0.75 Kolmogorov-Smirnov test Levene’s test LASSO	ML	LR	Yes
Yunpei Zhu 2022	Manual	2D	Darwin Research platform	shape first order second order	- / 1125	Pyradiomics	ANOVA mRMR LASSO	ML	LR	Yes

2D: 2-Dimensional, 3D: 3-Dimensional, LR: Logistic Regression, DL: Deep Learning, ML: Machine learning, SVM: Support Vector Machine, PCA: Principal Component Analysis, LASSO: Least Absolute Shrinkage and Selection Operator, ICC: Interclass Correlation Coefficient, DT: Decision Tree, GIMP: GNU Image Manipulation Program

### 3.3. Quality assessment

The 23 studies reached a mean RQS score of 12.64 with a minimum of 6 and a maximum of 19 out of 36 possible points. **Table 1** shows the RQS score of each study in the corresponding column. The individual ratings for each RQS item and each study are provided in **S1 Table**. The following items were evaluated in none of the studies: phantom study, imaging at multiple points, cut-off analyses, potential clinical application, cost-effectiveness analyses, open science, and data.

**Fig 2:**
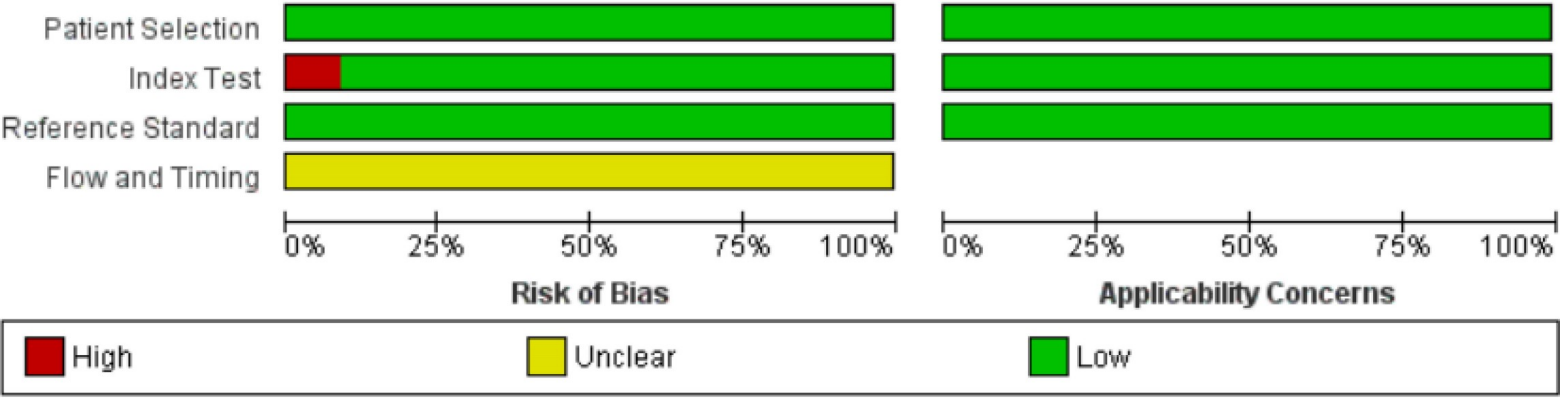
QUADAS-2 assessment of the included studies.

Details of the assessment of included studies using QUADAS-2 are shown in **S1 Fig** and **Fig 2** All studies assigned are “unclear” regarding the flow and timing item because none of the studies provided the timing of biopsy before US examination. Hao Cui et al. and Jia‑wei Li et al. assigned a “High risk of bias” because the imaging process approach and validation step were not specified.

### 3.4. Predictive performance of AI methods for HER2

Seventeen studies (17/23) predicted the HER2 status in BC patients, of which ten studies (10/17) had adequate data for quantitative analysis. Studies with insufficient data [15, 17, 18, 23, 27, 30, 37] were excluded from the quantitative analysis. For the prediction of HER2 status, pooled estimates were sensitivity, specificity, and summary receiver operating characteristic (SROC) with a 95% confidence interval. **Table 3** shows the categorized extracted data for quantitative analysis. According to the generated forest plot (**S2 Fig**), the pooled sensitivity and specificity were 0.76 [0.65, 0.84] and 0.78 [0.70, 0.84], indicating a high rate of heterogeneity for both sensitivity (I2 = 67.16 [45.31, 89.01]) and specificity (I2 = 91.41 [87.42, 95.40]). Pooled results from the AUC estimates were 0.84 [0.15, 0.99].

**Table 3:** Categorized extracted data for quantitative analysis.

Study	Molecule	Sensitivity	Specificity	Total	Pos vs. Neg	TP	FN	TN	FP	AI approach	Data availability
Fang Chen 2020 (Testing)	HER2+	0.7062	0.7897	39	20 vs 19	14	6	15	4	ML (texture)	Available
Hao Cui 2023	HER2+	0.9101	0.744	95	40 vs 55	36	4	41	14	Radiomics	ROC curve
Ioana Bene 2022 (Entire)	HER2+	0.7692	0.8475	72	13 vs 59	10	3	50	9	Radiomics	Available
Jia-wei Li 2022	HER2+	0.959	0.5	252	27 vs 225	26	1	112	113	Radiomics	Available
Meng-Yao Quan 2023 (IV)	HER2+	0.643	0.917	88	28 vs 60	18	10	55	5	DL-Radiomics	Available
Rong Xu 2023 (T)	HER2+	0.73	0.662	108	22 vs 86	16	6	57	29	Radiomics	Available
Xiaoying Zhuo 2023	HER2+	0.748	0.886	103	51 vs 52	38	13	46	6	DL	ROC curve
Xuantong Gong 2023 (Entire)	HER2+	0.465	0.816	110	31 vs 89	14	17	73	16	Radiomics	Available
Yinghong Guo 2022 (V-R)	HER2+	0.731	0.806	93	26 vs 67	19	7	54	13	Radiomics	Available
Romuald Ferre 2023 (Entire)	HER2+	0.714	0.716	66	21 vs 45	15	6	32	13	Radiomics	Available
Jiangfeng Wu 2022 (Test-R)	Ki67	0.887	0.708	86	62 vs 24	55	7	17	7	Radiomics	Available
Rong Xu 2023 (T)	Ki67	0.616	0.771	108	35 vs 73	22	13	56	17	Radiomics	Available
Jiangfeng Wu 2022 (Test-C)	Ki67	0.903	0.708	86	63 vs 24	56	6	17	7	Radiomics	Available
Jia-wei Li 2022	Ki67	0.6	0.864	252	195 vs 57	117	78	49	8	Radiomics	Available
Jinjin Liu 2022 (V-C)	Ki67	0.984	0.743	98	63 vs 35	62	1	26	9	Radiomics	Available
Yimin Wu 2023 (V-C)	Ki67	0.727	0.909	66	44 vs 22	32	12	20	2	Radiomics	Available
Yunpei Zhu 2022 (T-C)	Ki67	0.7	0.791	155	113 vs 42	79	34	33	9	Radiomics	Available
Mengyun Qiao 2022 (Entire)	Ki67	0.716	0.696	402	230 vs 272	165	65	189	83	DL	Available
Ioana Bene 2022 (Entire)	ER+	0.5593	0.8462	72	59 vs 13	33	26	11	2	Radiomics	Available
Rong Xu 2023 (T)	ER+	0.753	0.871	108	47 vs 68	35	12	59	9	Radiomics	Available
Ioana Bene 2022 (Entire)	PR+	0.4474	0.8824	72	38 vs 34	17	21	30	4	Radiomics	Available
Rong Xu 2023 (T)	PR+	0.786	0.731	108	52 vs 56	41	11	41	15	Radiomics	Available

TP: True Positive, FN: False Negative, TN: True Negative, FP: False Positive, IV: Internal Validation, ML: Machine Learning, DL: Deep Learning, ROC: Receiver Operating Curve, T: Testing, R: Radiomics model, C: Combined Model, V: Validation

The funnel plot did not show publication bias (p: 0.46) (**S3 Fig**). Regarding clinical utility, the Fagan plot (**S4 Fig**) showed that AI models of HER2 status would increase the post-test probability to 46% from 20% with a PLR of 3 when the pretest was positive and would reduce the post-test probability to 7% with an NLR of 0.31 when the pretest was negative.

Bo-Yang Zhou et al. [15] designed a prospective study showing that the US-based convolutional neural network (CNN) model has a high accuracy (AUC: 0.89–0.96). This study differs from the other studies by the type of prediction model that distinguishes four classes of molecular biomarkers (HER2, ER, PR, Ki67). For this reason, we did not include this study in the meta-analysis.

Meng Jiang et al. [17] designed a similar study to Bo-Yang Zhou et al. They constructed a deep-learning model of US images that differentiates the four categories of BC (Luminal A, Lumina B, HER2+, and Triple-negative) based on key molecular biomarkers. In two test sets, the model yielded a good accuracy of 80.07% to 97.02% and 87.94% to 98.83%. Compared to the study of Bo-Yang Zhou et al., this study used two test sets with accuracy similar to the mentioned study. The high level of accuracy may be due to the overfitting since both studies are deep-learning models.

Xianyu Zhang et al. [23] developed a deep learning US model for three-class classification of BC, including HER2, hormone receptor, and triple-negative. The deep learning model achieved an accuracy of 85.6%, with a gold standard of pathological results in the test set. Also, the model’s accuracy was 90% in an external test set. Interestingly, this study and the two previous [15, 17] studies with deep learning models had a high accuracy in test sets.

Similar to the previous studies, Zilong Xu et al. [30] constructed a deep learning US model. The model yielded an AUC of 0.84 and an accuracy of 80.56% in the validation set for HER2 status prediction.

Linyong Wu et al. [37] developed a US-radiomics model in patients with breast ductal carcinoma in situ (DCIS) to predict *HER2*, ER, PR, and Ki67. Since this study did not specify the exact number of positive and negative classes of each biomarker in training and testing sets, we did not include it in the quantitative analysis. Evaluations indicated that the model had an AUC of 0.94 and 0.74 in the training and test set for HER2 status prediction.

Mengwei Ma et al. [18] fitted the US texture features to different machine learning algorithms. They established different models for different tasks. All machine learning models yielded a similar AUC for differentiating HER2 from other subtypes. However, the Random Forest model performed slightly better, with an AUC of 0.855 in the test set. On the other hand, the logistic regression model outperformed other models to differentiate HER2+ from HER2- with a slightly better AUC of 0.744.

Taken together, AI models determined the HER2 status in BC patients with moderate to high precision in the quantitative analysis with a pooled sensitivity, specificity, and AUC of 0.76, 0.78, and 0.84, respectively. Deep learning models showed that outperformed the other AI methods for the prediction of HER2 status.

### 3.5. Predictive performance of AI methods for Ki67

Thirteen studies (13/23) predicted the Ki67 status in BC patients, of which seven studies (7/13) had adequate data for quantitative analysis. Studies with insufficient data were excluded from the quantitative analysis [15, 17, 18, 31, 33, 37].

For the prediction of Ki67 status, pooled estimates were sensitivity, specificity, and SROC with a 95% confidence interval. **Table 3** shows the categorized extracted data for quantitative analysis. According to the generated forest plot (**S5 Fig**), the pooled sensitivity and specificity were 0.80 [0.67, 0.89] and 0.76 [0.69, 0.81], indicating a high rate of heterogeneity for both sensitivity (I2 = 89.99 [84.49, 95.48]) and specificity (I2 = 62.76 [34.22, 91.30]). Pooled results from the AUC estimates were 0.81 [0.78, 0.84].

The funnel plot did not show publication bias (p: 0.09) (**S6 Fig**). Regarding clinical utility, the Fagan plot (**S7 Fig**) showed that AI models of Ki67 status would increase the post-test probability to 45% from 20% with a PLR of 3 when the pretest was positive and would reduce the post-test probability to 6% with an NLR of 0.26 when the pretest was negative.

Hao Cui et al. [31] examined the accuracy of a US radiomics model for Ki67 status prediction. Sensitivity and specificity are the two main metrics for evaluating diagnostic tools. This study did not report these two metrics and Ki67 classes in the dataset. However, the regression model achieved an AUC of 0.71.

In contrast to all other studies, Ioana Bene et al. [33] showed that no texture parameter was statistically significant in the univariate analysis for the Ki67 biomarker prediction in BC patients.

Also, Mengwei Ma et al. [18] developed different machine-learning models. In contrast to HER2 status prediction, the Naive Bayes (NB) model performed better than other algorithms. For differentiating high Ki67 from low Ki67 expression, the NB model had an AUC of 0.739 in the test set.

Altogether, AI models determined the Ki67 status in BC patients with moderate to high precision in the quantitative analysis with a pooled sensitivity, specificity, and AUC of 0.80, 0.76, and 0.81, respectively.

### 3.6. Predictive performance of AI methods for ER and PR

Six studies (6/23) predicted the ER and PR status in BC patients, of which only two studies (2/6) had adequate data [22, 33], which is not enough to pool the effect sizes for quantitative analysis. As mentioned, Bo-Yang Zhou et al. [15] designed a CNN model of US features with high accuracy (AUC: 0.89–0.96) in predicting BC molecular subtypes’ four-classification (HER2, *ER*, *PR*, Ki67). Also, Linyong Wu et al. [37] constructed a US-radiomics model to predict HER2, *ER*, *PR*, and Ki67 biomarkers. Analysis showed that the model had an AUC of 0.94 and 0.74 in the training and test set. We noted that Meng Jiang et al. [17] constructed a deep-learning model that could differentiate the four categories of BC (Luminal A, Lumina B, HER2+, and Triple-negative) based on key molecular biomarkers such as *ER* and *PR*. The model yielded a good accuracy of 80.07% to 97.02% and 87.94% to 98.83% in the two test sets.

Also, Mengwei Ma et al. [18] developed several machine-learning models. The LR algorithm showed the highest AUC of 0.878 among all machine learning algorithms for differentiating ER+ and ER- patients. Similarly, the LR algorithm outperformed others in differentiating PR+ and PR- patients with an AUC of 0.879.

We can conclude from the data that AI models of ER and PR status prediction have the potential to be applied in future clinical practice since models showed promising precision for differentiating ER and PR biomarkers in BC patients.

## 4. Discussion

This systematic review and meta-analysis showed promising diagnostic test accuracy for differentiating key molecular biomarkers of BC patients using AI methods such as radiomics and deep learning. The results of the quantitative analysis demonstrated moderate to high precision in differentiating key biomarkers of BC patients, especially HER2 and Ki67 molecules.

Data from the current analysis indicated that AI models could identify the status of HER2 and Ki67 biomarkers with an AUC of 84% and 81%, respectively. The current traditional method to determine the status of key molecular biomarkers in BC patients is mainly based on invasive procedures such as biopsy and excision samples of the tumor. Acquisition of tissue sampling using such local invasive methods can only capture a snapshot of the entire tumor since there is an intrinsic heterogeneity inside the tumor [9]. The new era of AI offers novel diagnostic methods for BC patients by exploring the tumor texture using mathematical and embedded algorithms and extracting quantitative distinct features non-invasively. In contrast to conventional methods, AI models not only explore the entire tumor instead of only a single part of the tumor but also do not lead to patient discomfort due to invasive procedures [10, 11].

This study meta-analysis supports the reliability of US-based AI models in differentiating HER2+ from HER2- with a sensitivity and specificity of 76% and 78%. Also, the analysis showed that US-based AI models could differentiate Ki67+ from Ki67- patients with a sensitivity and specificity of 80% and 76%. Regarding ER and PR markers, we could not pool the effect sizes and determine the accuracy of AI models quantitatively due to a small number of studies with adequate data for analysis. However, individual studies demonstrated promising diagnostic accuracy.

Breast US is the primary imaging modality to diagnose breast diseases since it is radiation-free, economical, real-time dynamic, tolerable by patients, and convenient for physicians. Meanwhile, the US has a relatively high accuracy for detecting breast diseases with different imaging features such as shape, margin, acoustic enhancement, vascularity, and calcification [38–40]. Thus, we emphasize the role of breast US as a reliable diagnostic test with valuable potential in detecting breast diseases, especially breast tumors, while highlighting the promising value of US to expedite the detection of key molecular biomarkers. Despite the promising findings of the quantitative analysis and the high accuracy of the models, there were some concerns about the methodological quality of the included studies. The QUADAS-2 tool indicated a low risk of bias in the patient selection, index test, and reference standard. However, no study reported the biopsy’s timing since tissue sampling could alter the texture features after the intervention. Also, studies showed no applicability concern for patient selection, index test, and reference standard.

Regarding radiomics studies, the RQS tool also proposed that the included literature has a moderate quality regarding RQS items. However, some ignored items by the studies deserve more attention. One of them is data and programming code sharing since it will increase the repeatability and producibility of the present data.

### 4.1. Limitations and future prospects

Although we reviewed the included studies comprehensively, this study encountered some inherent limitations. in this study, we included eligible studies regarding key molecular biomarkers in BC patients to pool the diagnostic effect sizes. However, several studies were short of enough data for quantitative analysis, which restricted us from reporting more reliable results, especially a single diagnostic metric for ER and PR markers. Therefore, we suggest future research provide data as far as possible to assist future meta-analyses in pooling the valuable findings to reach a single valuable finding. Only three studies retrieved the patients from multiple data centers. Since data collection from a single institute may not reflect the characteristics of the entire population, it is recommended to recruit patients from multiple data centers. Most of the included studies collected the data retrospectively. Since retrospective data collection has a high potential to cause bias, we need more prospective studies in the upcoming research. Since the assessment of the risk of bias and the quality of the literature showed some limitations, we are going to offer some recommendations: 1) Specify the selection criteria, study type, and patient characteristics; 2) Show the data acquisition, image processing, and validation process details; 3) apply the same reference standard to all patients as far as possible.

## 5. Conclusion

This meta-analysis indicated that AI models (radiomics, deep learning, machine learning) have a promising accuracy for predicting the status of key molecular biomarkers (HER2, Ki67, PR, ER) in patients with breast cancer. We performed the quantitative analysis for HER2 and Ki67 biomarkers which yielded a moderate to high accuracy. However, studies did not provide adequate data for meta-analysis of ER and PR prediction accuracy of developed models. The overall quality of studies, according to the QUADAS-2 and RQS tools, was acceptable. Considering the different US operators and machines, we suggest establishing standardized international protocols for performing ultrasound examinations, conducting interobserver agreement studies, and using standardized reporting systems. In future research in this field, we also need the studies to report the results thoroughly. Also, we suggest more prospective studies from different centers.

## Supporting information

S1 Table “TABLE S1” provides the RQS scores for individual studies. It also depicts the results of each study’s QUADAS-2 assessment and the forest plot, funnel plot, and fagan plot of the HER2 and Ki67 models.
(DOCX)

S1 Fig Results of QUADAS-2 assessment in the included studies. (JPG)

S2 Fig Forest plot of HER2 models. (PNG)

S3 Fig Funnel plot of HER2 models. (PNG)

S4 Fig Fagan plot of HER2 models. (PNG)

S5 Fig Forest plot of Ki67 models. (PNG)

S6 Fig Funnel plot of Ki67 models. (PNG)

S7 Fig Fagan plot of Ki67 models. (PNG)

S1 Checklist PRISMA checklist. 27-item checklist addressing different parts of systematic review and meta-analysis.
(DOCX)
