# Supplementary material for: Diagnostic performance of ultrasound-based artificial intelligence for predicting key molecular markers in breast cancer: A systematic review and meta-analysis
Source: PLoS One. 2024 May 31;19(5):e0303669. doi: 10.1371/journal.pone.0303669 (PMC11142607; doi:10.1371/journal.pone.0303669)
Supplement: S1 Table — It also depicts the results of each study’s QUADAS-2 assessment and the forest plot, funnel plot, and fagan plot of the HER2 and Ki67 models. (DOCX) [file pone.0303669.s001.docx]

**Table. S1**. RQS score of each radiomics study

|  | **Image Protocol  Quality** | **Multiple  Segmentation** | **Phantom  Study** | **Imaging At  Multiple Points** | **Feature  Reduction** | **Multivariable  Analyses** | **Biological  Correlation** | **Cut-off  Analyses** | **Discrimination  Statics** | **Calibration  Statics** | **Prospective  Study** | **Validation** | **Comparison to Gold Standard** | **Potential Clinical  Application** | **Cost Effectiveness  Analyses** | **Open science  And Data** | Total |
| --- | --- | --- | --- | --- | --- | --- | --- | --- | --- | --- | --- | --- | --- | --- | --- | --- | --- |
| Hao Cui 2020 | 1 | 0 | 0 | 0 | 0 | 0 | 1 | 0 | 1 | 0 | 0 | 0 | 3 | 0 | 0 | 0 | 6 |
| Hao Cui 2023 | 1 | 0 | 0 | 0 | 0 | 1 | 1 | 0 | 1 | 0 | 0 | 2 | 3 | 0 | 0 | 0 | 9 |
| Ioana Bene 2022 | 1 | 0 | 0 | 0 | 3 | 0 | 1 | 0 | 1 | 0 | 7 | 2 | 3 | 0 | 0 | 0 | 18 |
| Jiangfeng Wu 2022 | 1 | 1 | 0 | 0 | 3 | 1 | 1 | 0 | 1 | 1 | 0 | 2 | 3 | 0 | 0 | 0 | 14 |
| Jia‑wei Li 2022 | 1 | 1 | 0 | 0 | 3 | 0 | 1 | 0 | 1 | 0 | 0 | 2 | 3 | 0 | 0 | 0 | 12 |
| Jinjin Liu 2022 | 1 | 1 | 0 | 0 | 0 | 1 | 1 | 0 | 1 | 1 | 0 | 2 | 3 | 0 | 0 | 0 | 11 |
| Linyong Wu 2021 | 1 | 1 | 0 | 0 | 3 | 0 | 1 | 0 | 1 | 1 | 0 | 2 | 3 | 0 | 0 | 0 | 13 |
| Meng-Yao Quan 2023 | 1 | 0 | 0 | 0 | 0 | 3 | 1 | 0 | 1 | 0 | 0 | 2 | 3 | 0 | 0 | 0 | 11 |
| Romuald Ferre 2023 | 1 | 0 | 0 | 0 | 3 | 0 | 1 | 0 | 1 | 0 | 0 | 2 | 3 | 0 | 0 | 0 | 11 |
| Rong Xu 2023 | 1 | 1 | 0 | 0 | 0 | 0 | 1 | 0 | 1 | 0 | 0 | 2 | 3 | 0 | 0 | 0 | 9 |
| Xuantong Gong 2023 | 1 | 1 | 0 | 0 | 3 | 0 | 1 | 0 | 1 | 0 | 7 | 2 | 3 | 0 | 0 | 0 | 19 |
| Yimin Wu 2023 | 1 | 0 | 0 | 0 | 3 | 3 | 1 | 0 | 1 | 0 | 0 | 2 | 3 | 0 | 0 | 0 | 14 |
| Yinghong Guo 2022 | 1 | 1 | 0 | 0 | 3 | 3 | 1 | 0 | 1 | 0 | 0 | 2 | 3 | 0 | 0 | 0 | 15 |
| Yunpei Zhu 2022 | 1 | 0 | 0 | 0 | 3 | 3 | 1 | 0 | 1 | 1 | 0 | 2 | 3 | 0 | 0 | 0 | 15 |
