# Supplementary figures and images for: Diagnostic performance of ultrasound-based artificial intelligence for predicting key molecular markers in breast cancer: A systematic review and meta-analysis
Source: PLoS One. 2024 May 31;19(5):e0303669. doi: 10.1371/journal.pone.0303669 (PMC11142607; doi:10.1371/journal.pone.0303669)

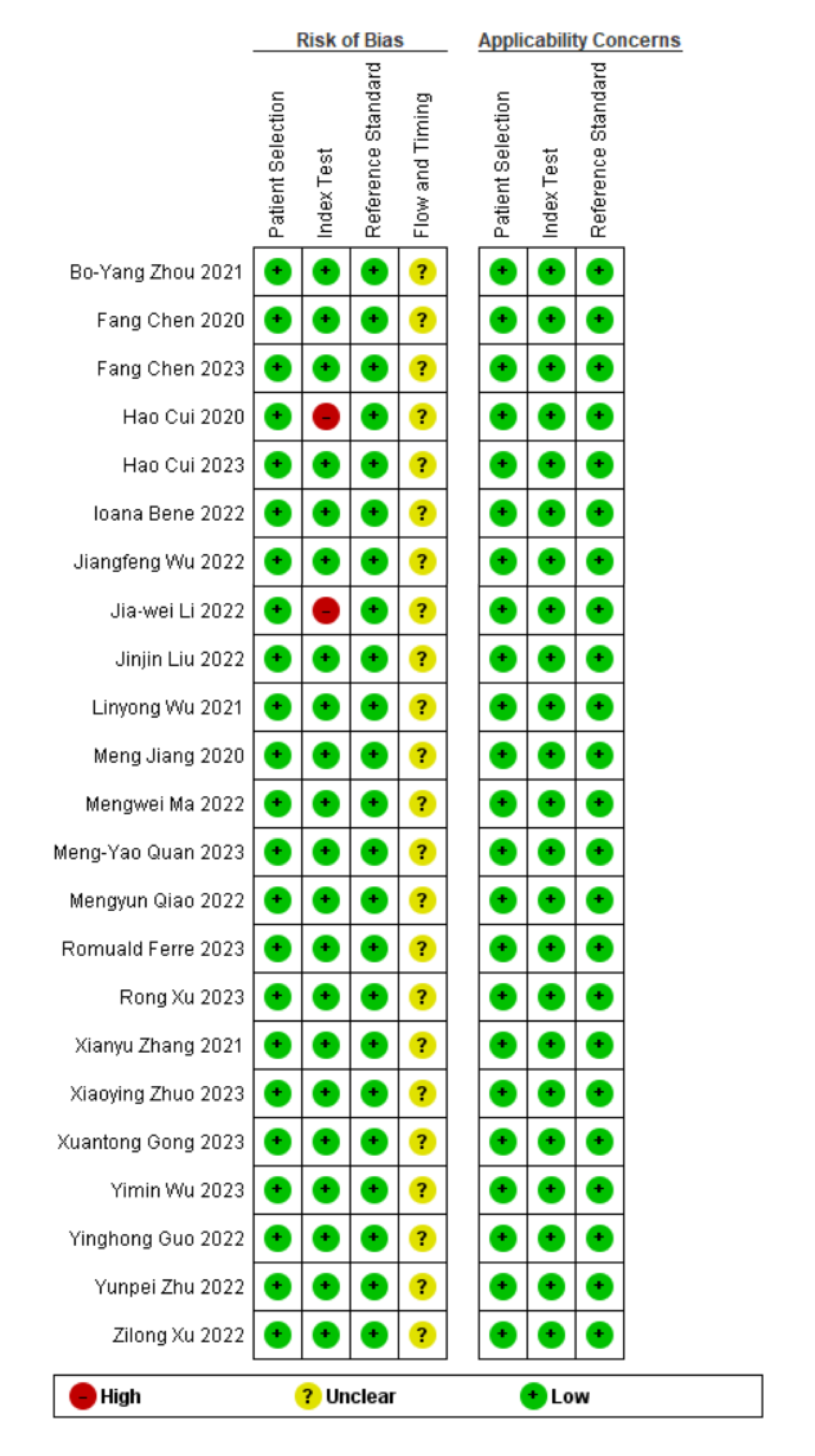

Supplement: S1 Fig — (JPG) [file pone.0303669.s002.jpg]

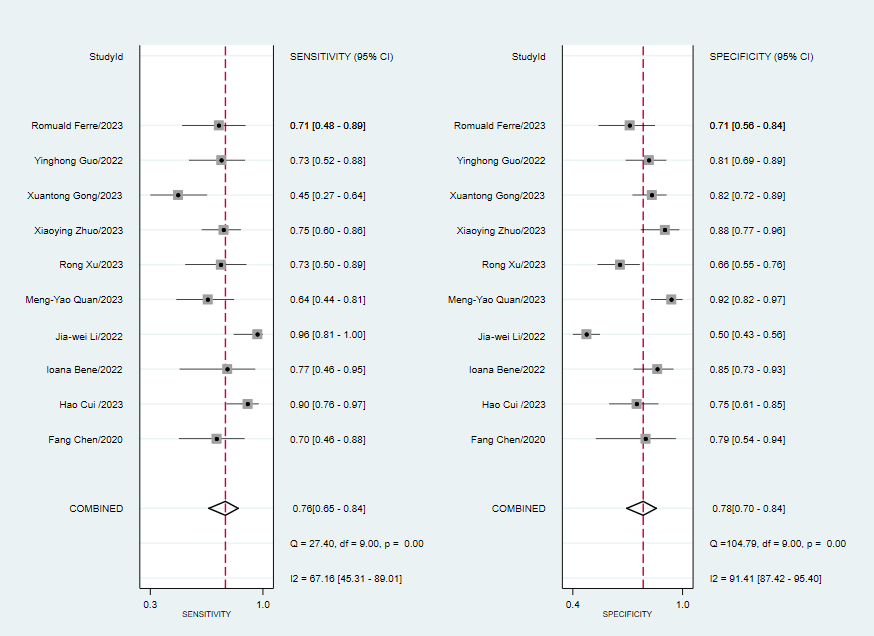

Supplement: S2 Fig — (PNG) [file pone.0303669.s003.png]

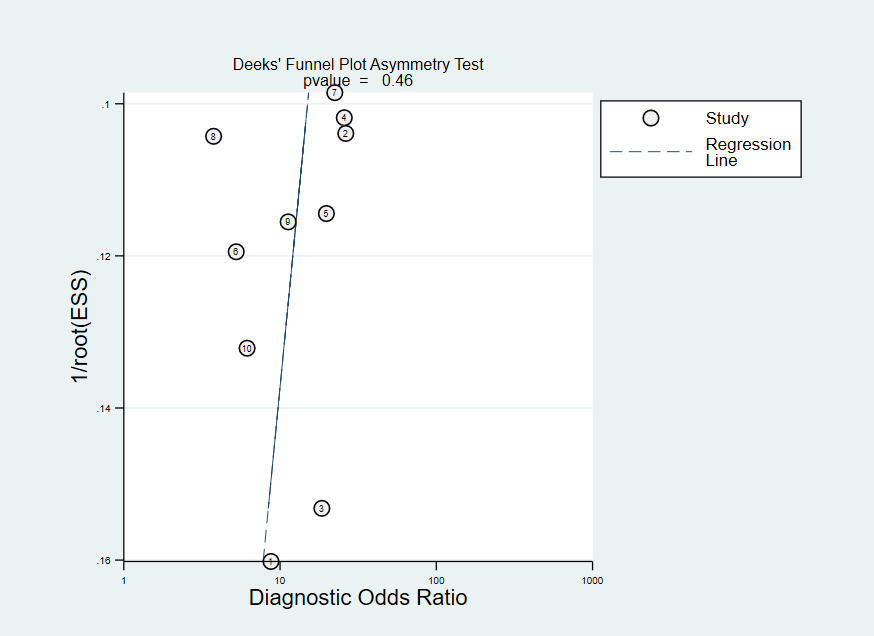

Supplement: S3 Fig — (PNG) [file pone.0303669.s004.png]

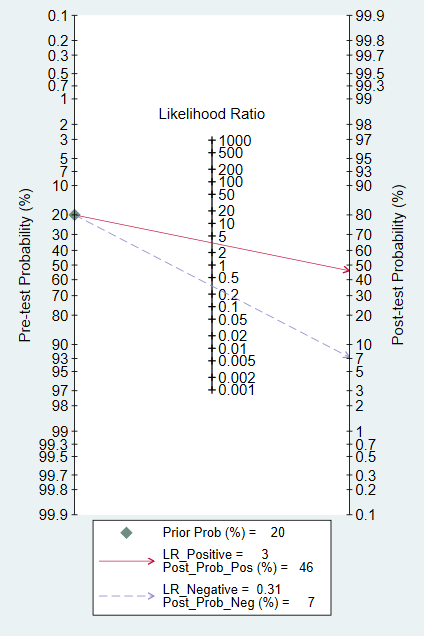

Supplement: S4 Fig — (PNG) [file pone.0303669.s005.png]

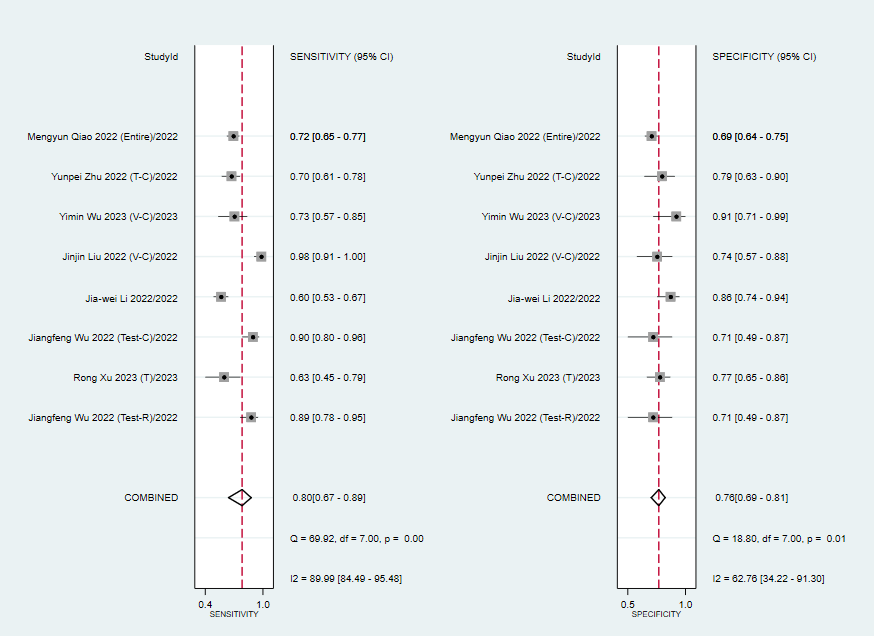

Supplement: S5 Fig — (PNG) [file pone.0303669.s006.png]

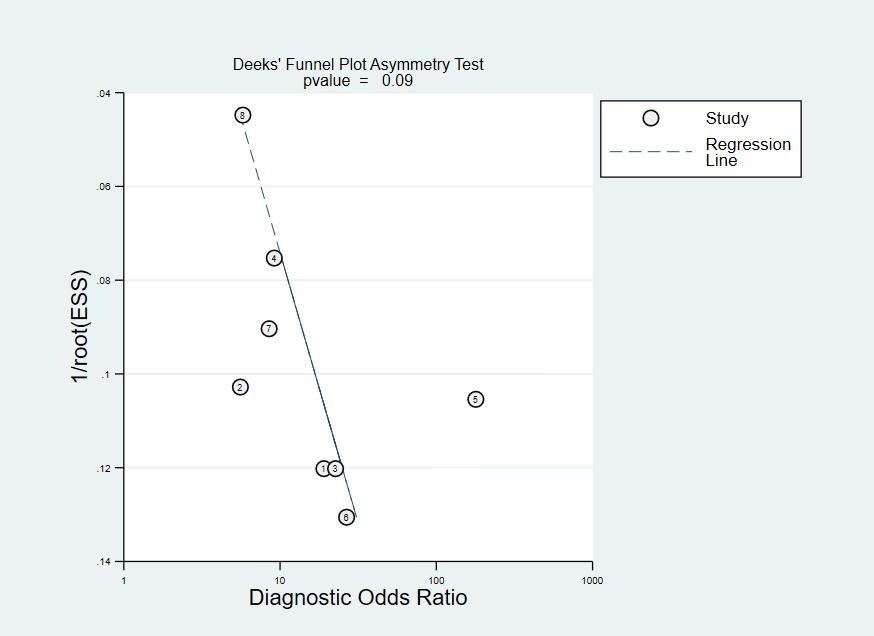

Supplement: S6 Fig — (PNG) [file pone.0303669.s007.png]

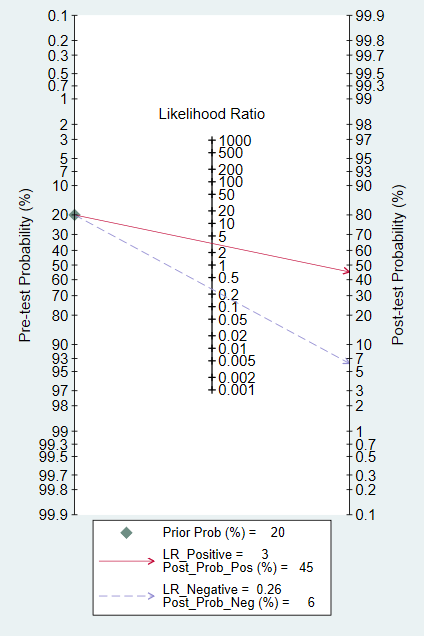

Supplement: S7 Fig — (PNG) [file pone.0303669.s008.png]
